# Is the detection of aquatic environmental DNA influenced by substrate type?

**DOI:** 10.1371/journal.pone.0183371

**Published:** 2017-08-16

**Authors:** Andrew S. Buxton, Jim J. Groombridge, Richard A. Griffiths

**Affiliations:** Durrell Institute for Conservation and Ecology, School of Anthropology and Conservation, University of Kent, Marlowe Building, Canterbury, Kent, United Kingdom; University of Hyogo, JAPAN

## Abstract

The use of environmental DNA (eDNA) to assess the presence-absence of rare, cryptic or invasive species is hindered by a poor understanding of the factors that can remove DNA from the system. In aquatic systems, eDNA can be transported out either horizontally in water flows or vertically by incorporation into the sediment. Equally, eDNA may be broken down by various biotic and abiotic processes if the target organism leaves the system. We use occupancy modelling and a replicated mesocosm experiment to examine how detection probability of eDNA changes once the target species is no longer present. We hypothesise that detection probability falls faster with a sediment which has a large number of DNA binding sites such as topsoil or clay, over lower DNA binding capacity substrates such as sand. Water removed from ponds containing the target species (the great crested newt) initially showed high detection probabilities, but these fell to between 40% and 60% over the first 10 days and to between 10% and 22% by day 15: eDNA remained detectable at very low levels until day 22. Very little difference in detection was observed between the control group (no substrate) and the sand substrate. A small reduction in detection probability was observed between the control and clay substrates, but this was not significant. However, a highly significant reduction in detection probability was observed with a topsoil substrate. This result is likely to have stemmed from increased levels of PCR inhibition, suggesting that incorporation of DNA into the sentiment is of only limited importance. Surveys of aquatic species using eDNA clearly need to take account of substrate type as well as other environmental factors when collecting samples, analysing data and interpreting the results.

## 1. Introduction

Environmental DNA (eDNA) is a rapidly expanding method for the detection and survey of aquatic organisms. Targeted species detection from samples of water using qPCR is increasingly being used in local and regional assessments of invasive[1], rare[2] or protected species[3]. The method is also being used to assess changes in site occupancy over time[3,4], where the use of traditional methodologies would be logistically onerous. For both national assessments and localised presence-absence surveys of target species it is important that limitations surrounding the technique and sampling strategy are understood. Indeed, where eDNA fails to detect a species that is known to have been recently present, understanding the persistence of eDNA is crucial for reliable interpretation of results.

Three processes contribute to the removal of eDNA from the aquatic environment, influencing the length of time a target organism can be detected. Firstly, transport in water flows in lotic systems[5] or currents in the marine environment[6]. However, this is unlikely in small lentic waterbodies such as ponds. Secondly, eDNA becomes unavailable for survey as the DNA is degraded through a wide variety of processes [7–11]. Thirdly, eDNA can be transported vertically out of suspension by binding to particulate matter, settling and becoming incorporated into substrates such as clay[8]. The number of binding sites and binding mechanisms within the substrate play a role in its capacity to bind with DNA, with sand having a lower capacity than clay due to particle size[12]. This difference in capacity means that substrate type can potentially alter the amount of DNA available in eDNA surveys. The persistence of aquatic eDNA is highly variable, with reports suggesting anything from a few hours[6] to two months[11] depending on environmental conditions. However, when incorporated into soil sediments, eDNA persistence may be in excess of months[13] or even thousands of years[14,15].

Where decreases in eDNA concentration are observed following the removal of the target organism, a pattern similar to a negative exponential decline has been documented[16–18]. In mesocosm experiments with Idaho giant salamanders (*Dicamptodon aterimus*), Pilliod *et al*.[17], show eDNA degradation of between 94% and 98% over the first two days, with the last positive samples found after 11 days. Also using mesocosms, Thomsen *et al*.[16] monitored eDNA persistence of the common spadefoot toad (*Pelobates fuscus*) and the great crested newt (*Triturus cristatus*), with detection persisting between 2 and 9 days. Neither study attempted to look at qPCR limits of detection or quantification[19]. It is therefore unclear whether the negative exponential decline is real or the studies merely reached their limits of quantification at the point where concentration decline appeared to slow.

Simply using the length of time during which eDNA remains detectable after the target species is removed does not show how the probability of detecting the species declines over time. Imperfect detection is commonplace within ecological studies causing errors within monitoring programs[20–26], and this is true for eDNA as well as conventional monitoring methods. Errors may arise during collection of the water sample, extraction of the DNA or amplification of the DNA. A false negative result (i.e., not detecting a species when in fact it is present) could result from non-uniform eDNA distribution within a waterbody, low concentration within the water body, degradation during sample transport and storage, PCR inhibition or poor affinity of the genetic assay with the target DNA[27,28].

PCR inhibition is common in environmental samples, with high concentrations of eDNA often being undetectable due to inhibitors[28]. There are many sources of PCR inhibition[29,30], and the cause of inhibition is not always apparent. However, humic substances from the breakdown of organic material[28] or derived from soils[29,31], are known to be PCR inhibitors Humic acids cause uncompetitive inhibition, binding to the polymerase active sites preventing the PCR reaction from occurring[29], reducing the efficiency of the PCR process and increasing the chance for false negative results.

The potential for false negative results therefore needs to be understood when using eDNA as a survey tool. Consequently, detection probability of eDNA–and how it changes over time–are important considerations. A number of studies report naïve detection rates based on the number of positives identified from a range of independent samples. In one well-studied species, the great crested newt, these naïve detection rates have been shown to vary widely from 60% to up to 99% [3,4,16,32], and this can lead to inconsistent–or even misleading–interpretation of the results.

Site occupancy detection models account for occasions when the sampling method may ‘miss’ the species (detectability) so that the proportion of sites in which the target species occurs (occupancy) can be reliably estimated [20,22,33]. When replicated samples are taken, the same principle can be applied to estimating the actual ‘occupancy’ of eDNA at a site. Indeed, occupancy models have been utilised for eDNA with a variety of taxa with the probability of detection ranging from 0.74 to 0.95[34–41].

In this study we utilise great crested newts, a semi-aquatic amphibian protected under UK and European legislation, as our study species. The species has been the subject of several eDNA studies[3,4,16,32,42], and eDNA surveys are now accepted practice in surveys of the species carried out as part of commercial development mitigation [43]. Using a mesocosm experiment with different pond substrates, we show how detection probability falls following the removal of the target species. We hypothesize that detection probability will reduce over time as eDNA becomes unavailable for the survey. We further predict this drop in detection probability will occur faster in water containing organic sediments or small particle size sediments than in water where no sediment is present or with large particle size inorganic sediments. Although changes in eDNA concentration and the proportion of amplifying replicates have been previously studied under semi-natural conditions [5,16], we believe that this is the first time occupancy models have been utilised in relation to mesocosm experiments to reliably determine changes in detection over time. Equally, we show for the first time how eDNA detectability varies in relation to sediment type.

## 2. Methods

### 2.1 Experimental set up

Twenty opaque PVC plastic boxes with a maximum volume of 20 L (width 36 cm x depth 28 cm x height 20 cm) were set up in a 5 x 4 grid in an outdoor field, with tanks separated from one another by approximately 30 cm. Each tank was randomly assigned one of four treatments, with five replicates of each treatment.

The four treatment groups comprised clay, sand, topsoil and a no substrate control group. Smooth terracotta potter’s clay was chosen to represent a substrate commonly used for pond lining, the substrate is 100% clay with impurities removed. Commercially available children’s play sand was used to emulate ponds with a sandy inorganic substrate. Commercially available garden centre topsoil was used to represent ponds with a high organic input. The topsoil consisted of 40% sand, 33.3% silt and 26.7% clay, identified using LaMottle Company soil texture test kit (following the manufacturer’s instructions)[44]. This is a similar composition to that found in ponds with a high leaf litter content (unpublished data). No substrate was added to the control treatment groups.

The commercially available substrates were tested for great crested newt DNA using a modified QIAamp Stool DNA Mini Kit (Qiagen^®^) extraction protocol and qPCR conditions described later (n = 8 replicates). Each of the plastic treatment boxes (except the control group) had 2.5 kg of the substrate added to it. Thirty litres of water were collected from each of eight high density great crested newt ponds[42], at the end of the breeding season on the 23^rd^ of May 2016 and mixed together in a large fiberglass tank to ensure a homogenous starting concentration. Five eDNA samples were collected at this stage to represent a baseline starting detectability and concentration. Ten litres of water were then transferred from the large fiberglass tank to each of the 20 treatment tanks. Opaque plastic lids were added to each treatment tank to prevent rainfall having a dilution effect or the effect of UV on eDNA breakdown. This was considered appropriate in the case of these mesocosms, because of the shallow nature of the water in each tank, UV would have penetrated the majority of the water and had a disproportionate influence compared to a natural pond. eDNA samples were collected from each of the 20 tanks 1, 2, 3, 4, 7, 9, 11, 14, 18 and 22 days after the water had been removed from the ponds.

### 2.2 Environmental covariates

Various environmental covariates were collected during the course of the study. pH, total dissolved solids and electro-conductivity were measured in each tank at the end of the study using electronic “pen type” meters (Hanna^®^ Instruments HI-98312 and AZ^®^ Instrument, 8685 pH Pen) following manufacturer’s instructions[45,46]. It was believed that these would not change considerably over the course of the study and the benefit of monitoring these daily was outweighed by the risk of contamination of target DNA between tanks. Air temperature was logged hourly at the site using Tinitag^®^ Plus2 –TGP-4017 (Gemini Data Loggers, Chichester, UK).

### 2.3 eDNA sample collection protocol

eDNA samples were collected using the precipitation in ethanol approach as developed by Biggs (*et al*.*)* [3]. Six 50 mL centrifuge tubes (Corning, Centristar^™^ Cap, 430828) containing 33 mL of absolute ethanol and 1.5 mL of 3M sodium acetate solutions, made up one sample. Using a sterilised disposable plastic pipette, each of the six centrifuge tubes was filled to the 50 mL gradation with water directly from the middle of the water column of the tank without stirring. This provided a total sample volume of approximately 90 mL. Samples were immediately stored at -20°C until extraction, this both aided sample preservation as well as the precipitation of DNA out of solution.

### 2.4 Laboratory protocol

eDNA sample extraction was undertaken using a modified Qiagen^®^ DNeasy^®^ Blood and Tissue kit protocol. A sample was removed from the freezer and centrifuged at 11000 RPM (14069g) for 30 minutes at 6°C. The supernatant was poured off leaving a pellet containing DNA and other matter that had precipitated out of solution on the side of each tube. The pellet from the first tube was suspended in 360 μL ATL buffer from the Qiagen^®^ DNeasy^®^ Blood and Tissue kit by vortexing for several minutes, the buffer solution containing the re-suspended pellet was then transferred to the second tube and the process repeated until each tube had been sequentially vortexed, and all six pellets suspended in the same solution. The ATL buffer solution was then transferred to a 2 mL microcentrifuge tube, 25 μL of ProK added and samples incubated at 56°C overnight. Extraction continued as per extraction kit manufacturer’s protocol, with spin columns eluted twice with 100 μL of warm AE buffer. Periodic extraction negative control samples were run through the course of the project.

Each sample was tested for PCR inhibition using TaqMan^®^ Exogenous Internal Positive Control (IPC) Reagents (Applied Biosystems^™^), following manufacturer’s instructions, with TaqMan^®^ Environmental Master Mix 2.0 (Applied Biosystems^™^). Samples were identified as inhibited if the IPC failed to amplify or late amplification (amplification outside 1 qPCR cycle from the qPCR negative control samples) was observed within the internal positive control.

qPCR was undertaken on all samples whether inhibited or not following the assay and conditions from Biggs (*et al*.*)*[3], using *Triturus cristatus* PCR primers TCCBL, TCCBR and hydrolysis probe TCCB developed by Thomsen *et al*.[16]. qPCR was conducted using a BioRad Laboratories, CFX Connect^™^ Real-Time PCR Detection System. qPCR was repeated on each sample eight times[42]. Each qPCR plate contained three standards for quantification, each repeated three times, as acting as positive controls, and three PCR negative controls. qPCR standards were made up of a dilution from great crested newt tissue extract and were quantified using a Qubit^®^ 2.0 with the Qubit^®^ dsDNA high sensitivity assay (Life Technologies^™^) at concentrations of 12.500 ngμL^-1^, 1.140 ngμL^-1^ and 0.120 ngμL^-1^, qPCR R-squared values ranged between 0.994 and 0.999, with a mean efficiency of 85.5%. A replicate was deemed to be positive if an exponential growth phase was observed during qPCR. The median concentration of the eight qPCR replicates was utilized as the concentration for a sample in analysis. During qPCR all negative control samples showed no deviation from the baseline, and were therefore clear negatives. Limits of detection and quantification were generated as per Buxton *et al*. [42].

Due to high levels of inhibition within the topsoil treatment group (see Results), all topsoil samples were treated as potentially inhibited. A 1 in 10 dilution using ddH_2_O was then undertaken on inhibited samples, to attempt to remove inhibitors and improve detection[27–29,47–50]. The diluted samples were then re-run using the internal positive control and qPCR protocol outlined above[4,51,52]. Trials using Bovine Serum Albumin (BSA) and lower dilution levels were undertaken but failed to remove inhibitors sufficiently (data not presented).

### 2.5 Analysis

As eDNA concentrations often fall below the limit of quantification achieved by qPCR, the use of eDNA concentration within the analysis would only be of limited value. However, occupancy modelling can be used to generate the probability of detection, independently of the concentration within a sample. Single season occupancy models were constructed based on single eDNA samples (representing ‘sites’ in traditional occupancy modelling) and repeated qPCR runs (representing observations). Models were constructed using R version 3.3.2[53] (with package Unmarked version 0.11–0[54], to observe the influences on detection probability across the study. Models were constructed using the occu function, with variable detection but constant occupancy. Site covariates, included in the analysis were substrate type, days since removal of target species and tank pH. Model selection was undertaken utilising the inbuilt model selection option within the Unmarked package. Models were ranked using the Akaike Information Criterion (AIC) and were weighted to indicate relative support of a model. Models with ΔAIC < 2 had strong support while models with a ΔAIC of >2 were considered to have less support[55]. Detection probabilities were then generated, using the predict function within the unmarked package and the model containing day and substrate variable detection.

We observed the rate at which the detection probability changed each day (Δ*p*/day) by taking the difference between predicted detection probabilities from one day to the next, for each of the sediment types. We examined whether maximum, minimum and mean external temperature influenced detection probability or Δ*p*/day, with generalised linear models (GLM) using R version 3.3.2[53].

### 2.6 Ethical assessment

The experimental procedure was approved by the University of Kent, School of Anthropology and Conservation, Research and Ethics committee. All sampling was undertaken from water and no animals were used as part of this work. Positive control samples within PCR were set up from DNA extracts from a long deceased great crested newt held under licence from Natural England licence number 2015-7591-SCI-SCI-1.

## 3. Results

### 3.1 Degradation

The commercially available sediments all tested negative for great crested newt DNA. In the clay, sand and control treatments eDNA concentration fell from 0.00108 ngμL^-1^, the mean concentration found on day 0, to the limit of quantification of 0.00005 ngμL^-1^ by day 4, a decrease of over 95%. In the topsoil treatment, eDNA concentration fell faster, reaching the limit of quantification between days 2 and 3. Beyond day 4 most samples fell below the limit of quantification for qPCR and so no accurate analysis can be undertaken with regard to eDNA concentrations. Samples were first observed as negative in the topsoil treatment group on day 7, in clay on day 14, and in sand and the control on day 18.

### 3.2 Detection probability

Models were included to predict what was influencing detection probability. The model with most support included detection based on number of days since the species was present (estimate = -0.320; z = -20.56; SE = 0.0155; p<0.0001), pH (estimate = -0.171; z = -1.66; SE = 0.1030; p = 0.0974) and substrate type with constant occupancy (Table 1). Although pH was included in the top model, it was not found to be significant. There was a significant reduction in detection in the topsoil treatment (estimate = -0.850; z = -3.85; SE = 0.2207; p = 0.000116), compared to the control group. However, no significant difference in detectability was found between the control group and both the clay treatment (estimate = -0.374; z = -1.83; SE = 0.2045; p = 0.0673) and the sand treatment (estimate = -0.003; z = -0.014; SE = 0.2053; p = 0.989). All covariates included within all models with a ΔAIC of <2 with the exception of substrate type and day were found not to be significant.

**Table 1:** The models with most support based on AIC criterion and AIC model selection. Top three models and all models with a ΔAIC of <2 presented. All models contain variable detection rates but constant occupancy. Days since the target species was in contact with the water, pH within each mesocosm and substrate treatment group were the only covariates found to be in the three models with most AIC support. nPars represents the number of parameters in the model.

Model	nPars	AIC	ΔAIC	AIC weight	Cumulative weight
*Constant occupancy*, detection variable by day, substrate and pH	7	1244.73	0.00	0.59	0.59
*Constant occupancy*, detection variable by day and substrate	6	1245.48	0.75	0.41	1.00
*Constant occupancy*, detection variable by day	3	1259.15	14.43	<0.01	1.00

**Fig 1:**
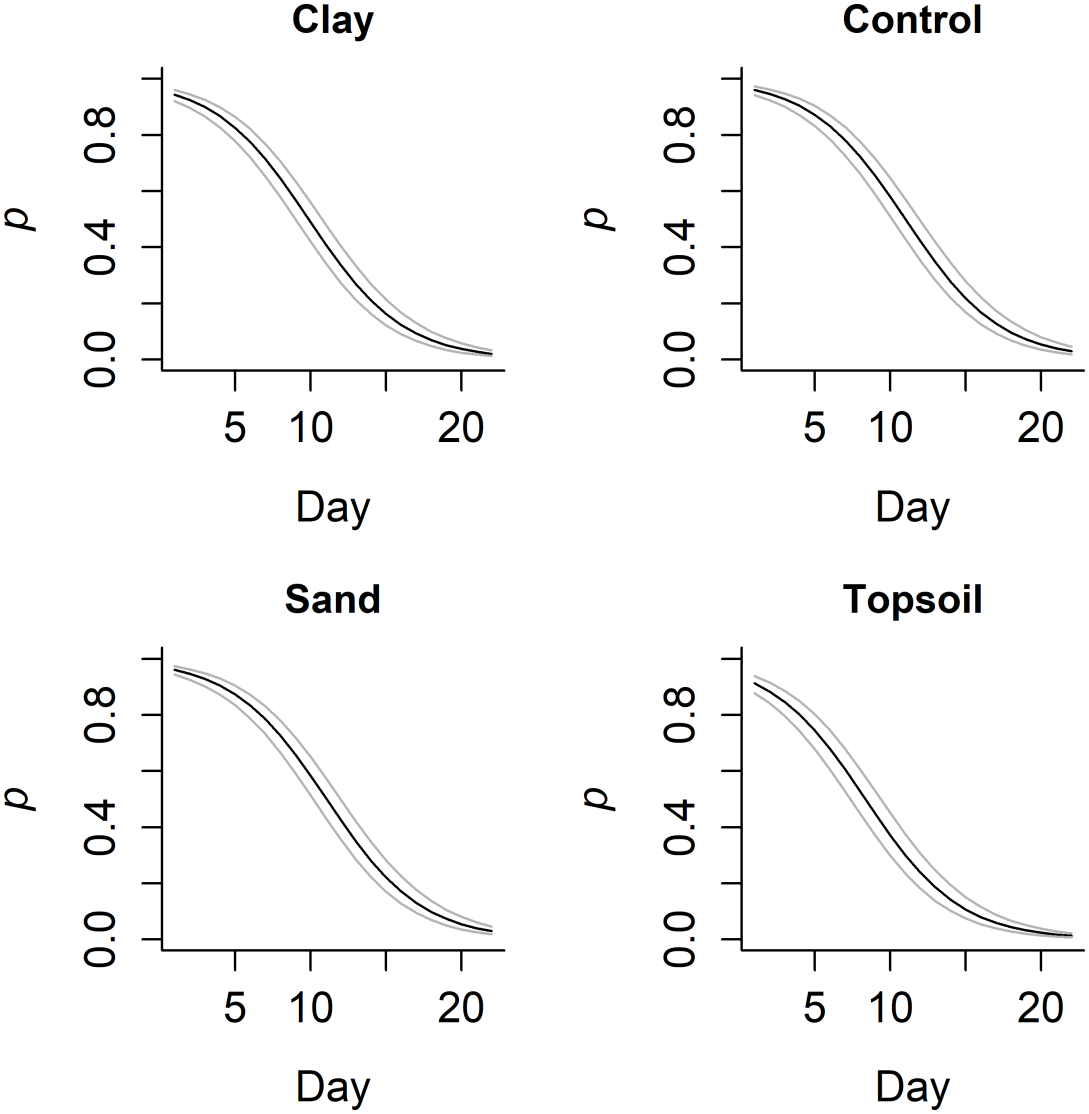
Decline in eDNA detection probability (*p*) over time, using eight qPCR runs per sampling occasion, following the removal of the target species from the water, with different substrate types. Light grey lines show 95% confidence limits.

The model with the second highest support included constant occupancy, but variable detection based on substrate type and day was used to predict detection probability in the different substrate types and across the study (Fig 1). The model with the most support was not used because the pH covariate was insignificant and would therefore have only confused the predictions. Detection probability (*p*), based on replicated PCR runs, was initially very high with sand and control treatment groups with *p*>0.96, and the clay treatment group *p* = 0.94. The topsoil treatment group showed a reduced starting detection probability at *p* = 0.91 (Fig 1). Detection fell slowly for the first few days, and by day five detection probability had fallen to *p* = 0.87 in the control and sand treatments, *p* = 0.83 for clay and *p* = 0.75 for topsoil treatment. Detection rate then fell more sharply from *p* = 0.58 for the control and sand treatments by day 10 and *p* = 0.22 by day 15: this was more pronounced in the clay and topsoil treatments where detection fell more rapidly to *p* = 0.49 and *p* = 0.37 respectively by day 10 and *p* = 0.16 and *p* = 0.10 respectively by day 15. By day 20 detection probability had fallen to 0.05 or below in all treatments (Fig 1).

Samples collected from tanks with a topsoil substrate were more likely to contain PCR inhibitors with 70% (35/50) of the samples showing signs of inhibition, compared to 2% (1/50) in the clay treatment group and no samples from the sand or control groups. Samples treated for the removal of inhibitors were found to all be free from inhibitors; however, a drastic reduction in detection probability was observed, from *p* = 0.91 in the original samples to p = 0.39 in the same samples when diluted to remove inhibitors. As a result the diluted data were discarded and analysis undertaken on the inhibited but undiluted data.

**Fig 2:**
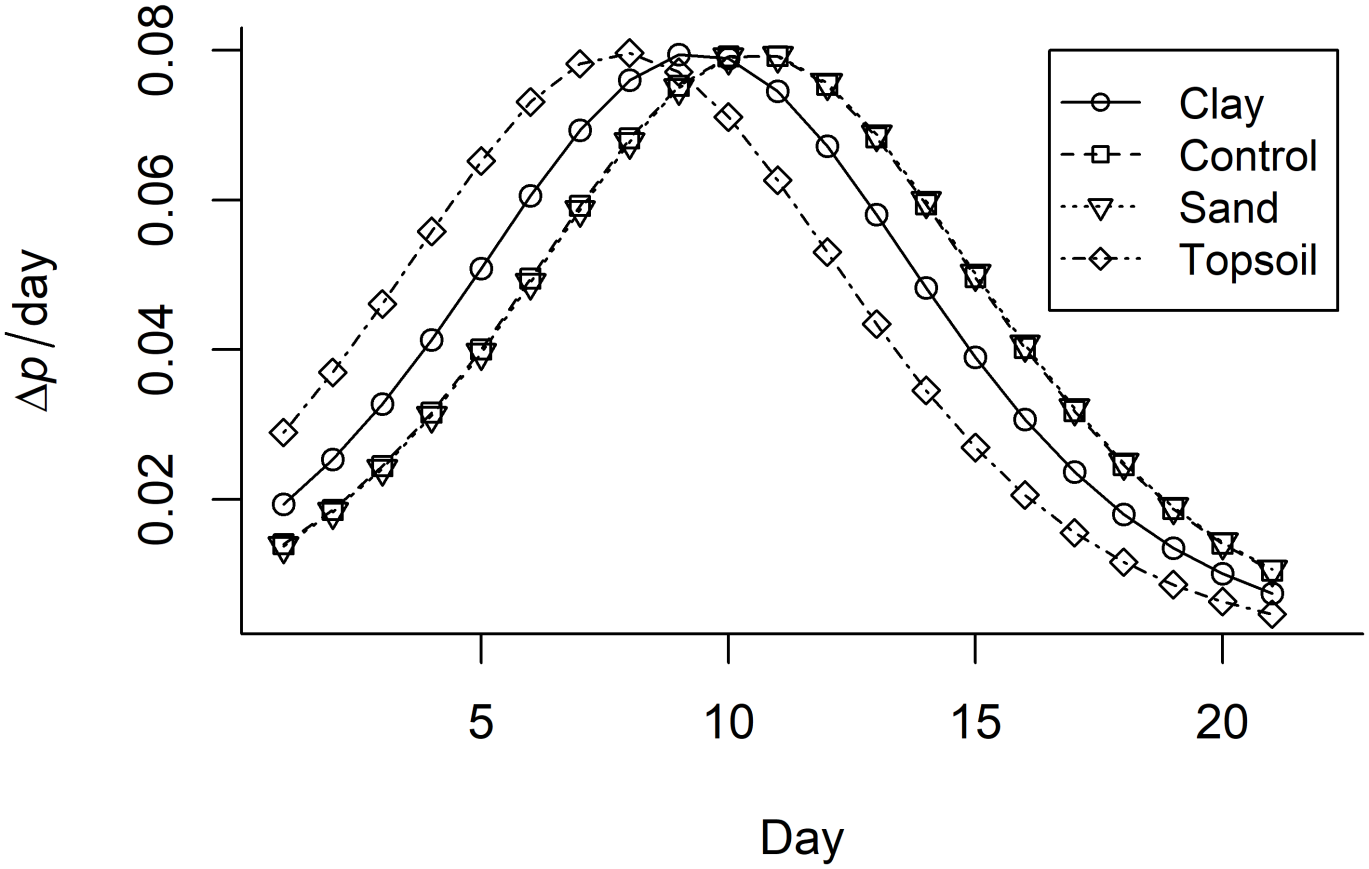
The rate of change in eDNA detection probability (*p*) each day in mesocosms with four sediment types.

The change in detection probability per day (Δ*p/day*) (Fig 2), is initially low for all treatment groups, increasing towards the middle of the study before reducing in the latter stages. No difference was observed between sand and the control treatment groups, initially at approximately 0.015 Δ*p/*day, increasing to a peak of 0.08 Δ*p/*day by day 11, before falling again until the end of the study. Rate of detection initially decreased more in the clay and topsoil treatments (0.02 and 0.03 Δ*p/*day respectively) than in the control treatment group. Both reached a peak rate of change of 0.08 Δ*p/*day on days 9 (sand) and 8 (topsoil). The rate of change for sand and topsoil then started to reduce earlier in the study than the control or clay treatment groups, and continued to reduce through the rest of the experiment.

A general linear model was used to assess whether the external temperature influenced the rate of changed in detection. A negative relationship between maximum air temperature and change in detection probability was identified, with greater rate of change at lower temperatures (S1 Fig). This was found to be significant for all four sediment types (control: estimate = -0.0024, SE = 0.0007, t-value = -3.325, p-value = 0.0038; sand: estimate = -0.0024, SE = 0.0007, t-value = -3.309, p-value = 0.0039; clay: estimate = -0.0023, SE = 0.0007, t-value = -3.433, p-value = 0.0030; topsoil: estimate = -0.0018, SE = 0.0006, t-value = -3.862, p-value = 0.0104). Mean daily temperature was not found to be significant for any of the treatment groups, and minimum temperature was only found to have a significant (positive) influence on the topsoil treatment group (estimate = 0.0049, SE = 0.0020, t-value = 2.411, p-value = 0.0268). This is surprising given that increases in temperature are linked with increases in DNA degradation rate and this is likely to be a coincidental artefact of the weather during the course of the experiment rather than an overriding influence on the change in detection probability.

## 4. Discussion

If a species vacates a waterbody, detection of that species remains possible using eDNA. Detection when a waterbody is no longer occupied is a distinct advantage over traditional survey methods. We have shown that the probability of detecting a species decreases with time, following its removal; however, the rate at which the probability of detecting the species decreases is not constant. We have not only shown that detection probability of eDNA is dependent on time since the organism was present, but we also show that the type of sediment influences detectability and the rate at which detectability decreases.

The initial detection probability for eDNA was very high—between 91% and 95%—the exception being where samples had been diluted to remove inhibitors. This high detection probability may have been because the water was sourced from small ponds with a very high target species density, and collected at the end of the breeding season when eDNA concentration is high[42]. As a result, the amount of DNA within the water was likely to be higher than that found in more typical, natural ponds or at other times of year[42]. There is a discrepancy between the results of our study and those of Thomsen *et al*.[16] in which eDNA was only detected for nine days—compared to 22 days in our study. Both studies targeted the same sequence in the cytochrome b gene and used the same target species. This discrepancy may be down to differences in both initial concentration of target DNA and collection methods used, with our study collecting a sample volume six times greater than Thomsen *et al*.[16].

The reduction in detection over time is likely due to the removal of target DNA through both degradation[9] and vertical transport and incorporation into the sediment[8]. The rate of change in detection was initially slow, an increase in the middle part of the study was observed peaking at approximately 0.08 Δ*p/*day in all treatment groups, and a reduced rate at the end. This pattern was observed in all sediment types. Reduced rate of change towards the end of the study may represent a slowing in the rate of degradation at lower eDNA concentrations, as at lower concentrations the chance of DNA molecules being broken down by DNase enzymes is reduced[12].

Detectability and the rate of change in detectability varied between the sediment types. It is likely that the type and quantity of PCR inhibitors released into the water differs between sediment types[30,31]. Soil structure may also influence the capacity for DNA to become incorporated into the different sediments[12]. No differences were observed in detectability or rate of change in detectability between the sand and control groups. However, we observed both a lower starting probability of detection and an increase in the initial rate of fall in detectability within the topsoil compared to the control treatment group. There was a tendency for a reduction in probability of detection in the clay treatment group compared to the control and sand treatment groups, although this was not significant.

PCR inhibitors interact either with DNA or DNA polymerase and can result in an increase in the number of cycles required to observe amplification of the target DNA over uninhibited samples; additionally reductions in the number of qPCR repeats which amplify, inconsistent amplification of the qPCR repeats or complete failure to amplify any target DNA may be observed[29,30]. PCR inhibitors are common within environmental samples and strong seasonality in inhibition has been observed and linked with the accumulation and degradation of leaf litter[28], consistent with high organic content of topsoil. PCR inhibition led to a drop in detection probability for the topsoil treatment group over the other sample groups, as well as lower extract concentration being observed in qPCR due to late amplification.

Initially, total dissolved solids (TDS) within each tank was included in in the analysis. However, TDS value was found to be significantly dependent on substrate type (S2 Fig) and so the two factors are not independent. TDS may therefore increase in suspended solids within the water column, rather than within the sediment itself. This may be responsible for the difference in PCR inhibition seen between the treatment groups: 0% of samples inhibited in control and sand, 2% of samples inhibited in the clay group and over 70% of samples inhibited in the topsoil treatment group. TDS within the topsoil treatment group was by far the highest and it is likely that PCR inhibitors within the dissolved solids such as humic acid[29,30,56] were the cause of the reduction in detection probability over the other treatment groups.

The dilution of the topsoil samples to remove inhibitors led to a 52% reduction in detection probability and therefore an increase in false negative results, as the DNA was diluted to undetectable levels[28,29,57]. We therefore argue that dilution approaches for the removal of inhibitors from eDNA samples compromises detection probability: other methods which do not result in a dilution of target DNA should therefore be explored.

In addition to inhibition, the rate at which DNA is incorporated into the sediment may cause the availability of eDNA within the water column to vary[8]. eDNA can become incorporated into substrates and absorbed onto minerals, binding to both humic compounds and soil minerals[12]. Little difference was identified in detection between the control group and the sand treatment group. Sand has a very large particle size, which results in a lower surface area and fewer binding sites than substrates with smaller particle size such as clay[12]. As a result, more DNA would be expected to remain detectable in water with a sandy substrate, than with a clay or topsoil substrate. Humic substances likely to be found within the organic topsoil also provide key binding sites for DNA[12]; this may reduce the availability of the target DNA for survey. However, it is likely that the greatly reduced detection probability in the topsoil treatment group results from a combination of an increase in PCR inhibitors[29], as well as removal of available target DNA from the water column.

eDNA research is still an evolving discipline. Unlike more widely recognised survey methods for freshwater species, the influences on and limitations of detection are still being identified. Our findings have important implications for how eDNA results are analysed and interpreted. Although detection of eDNA does not necessarily correspond to the concurrent presence of the species, the chance of detecting the species after it has vacated a pond reduces rapidly, and after three weeks can be as low as just 3.9%, as observed in our control group. To maximise the chance of detection, it is therefore advisable to collect samples when the target species is likely to be present, to minimise the chance of false absences. Pond specific characteristics such as the sediment also influence the probability of detecting the target organism, either by increasing PCR inhibition or through other mechanisms. It is therefore important to recognise when planning or interpreting the results from an eDNA study, that sediment has an influence of the efficacy of the survey method, and ponds with organic sediment types—or sediments that become suspended easily—can be a source of false negative results.

## Supporting information

S1 Fig Mean, maximum and minimum air temperature over the course of the study. Day one temperatures relate to the 24 hours leading up to the sampling time on the 24^th^ of May 2016.
(TIF)

S2 Fig Difference in total dissolved solid (TDS) levels within the mesocosms of different substrate types. Showing the median values with interquartile ranges. An analysis of variance yielded significant variation between sediment type and the TDS loading (F = 2464, DF = 3, 16, 16; p<0.0001). A post-hoc Tukey test showed no significant difference between the control group and sand (p = 0.98) but all other pairs had highly significant differences (p<0.0001).
(TIF)

S1 Data Spreadsheet containing the raw data including, detection/non-detections for qPCR replicates, derived concentrations from qPCR, and presence of inhibition within each sample as well as environmental covariates. (CSV)
