# Supplementary figures and images for: Is the detection of aquatic environmental DNA influenced by substrate type?
Source: PLoS One. 2017 Aug 16;12(8):e0183371. doi: 10.1371/journal.pone.0183371 (PMC5558973; doi:10.1371/journal.pone.0183371)

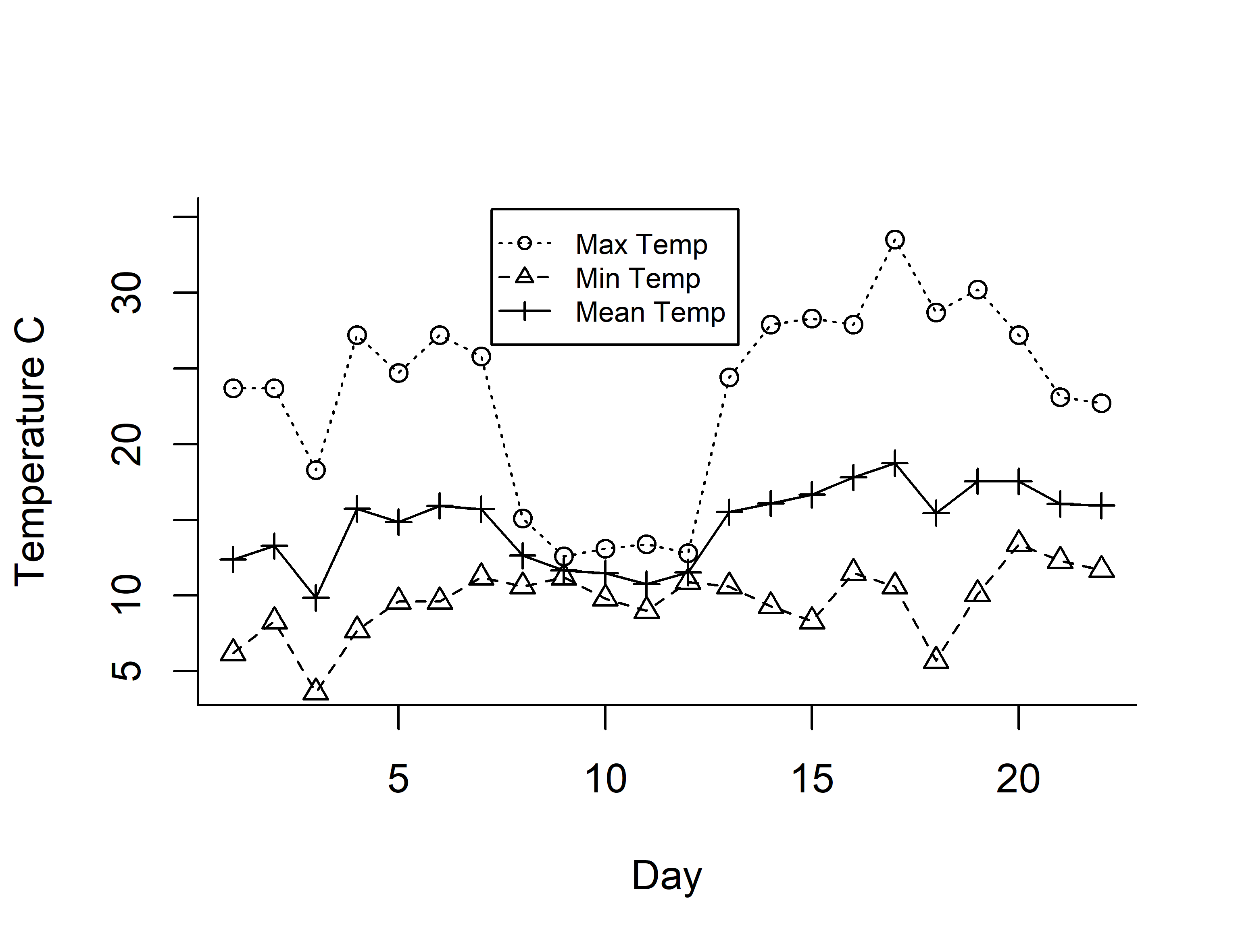

Supplement: S1 Fig — Day one temperatures relate to the 24 hours leading up to the sampling time on the 24th of May 2016. (TIF) [file pone.0183371.s001.tif]

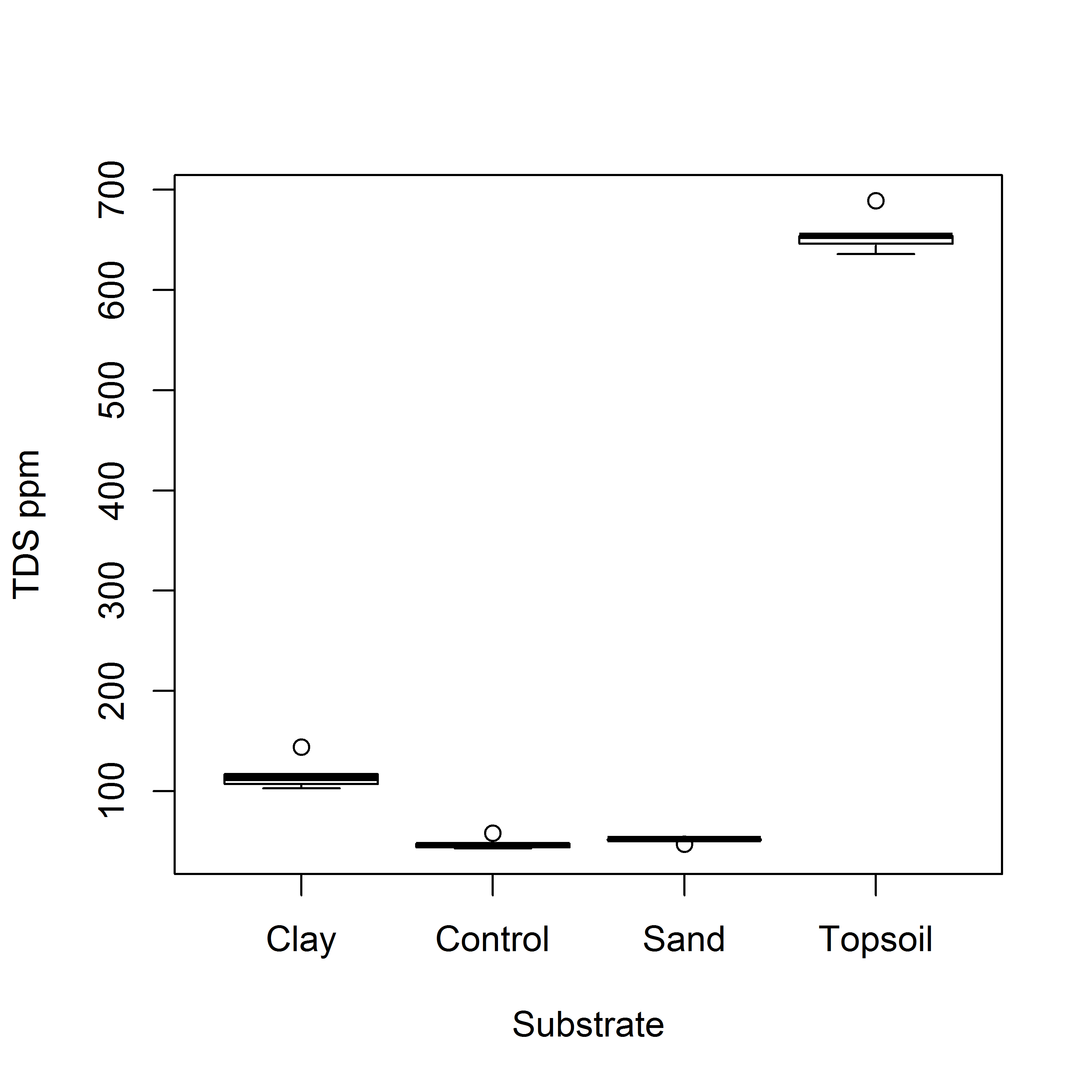

Supplement: S2 Fig — Showing the median values with interquartile ranges. An analysis of variance yielded significant variation between sediment type and the TDS loading (F = 2464, DF = 3, 16, 16; p<0.0001). A post-hoc Tukey test showed no significant difference between the control group and sand (p = 0.98) but all other pairs had highly significant differences (p<0.0001). (TIF) [file pone.0183371.s002.tif]
